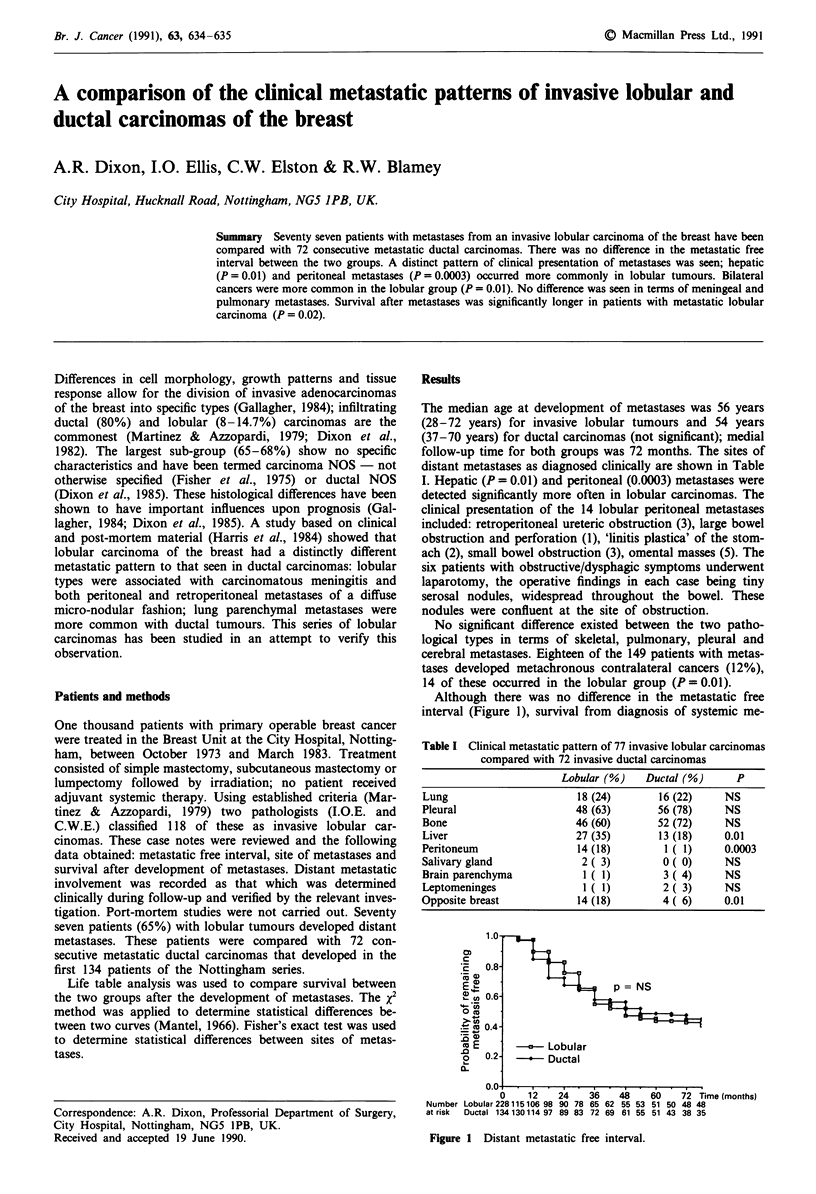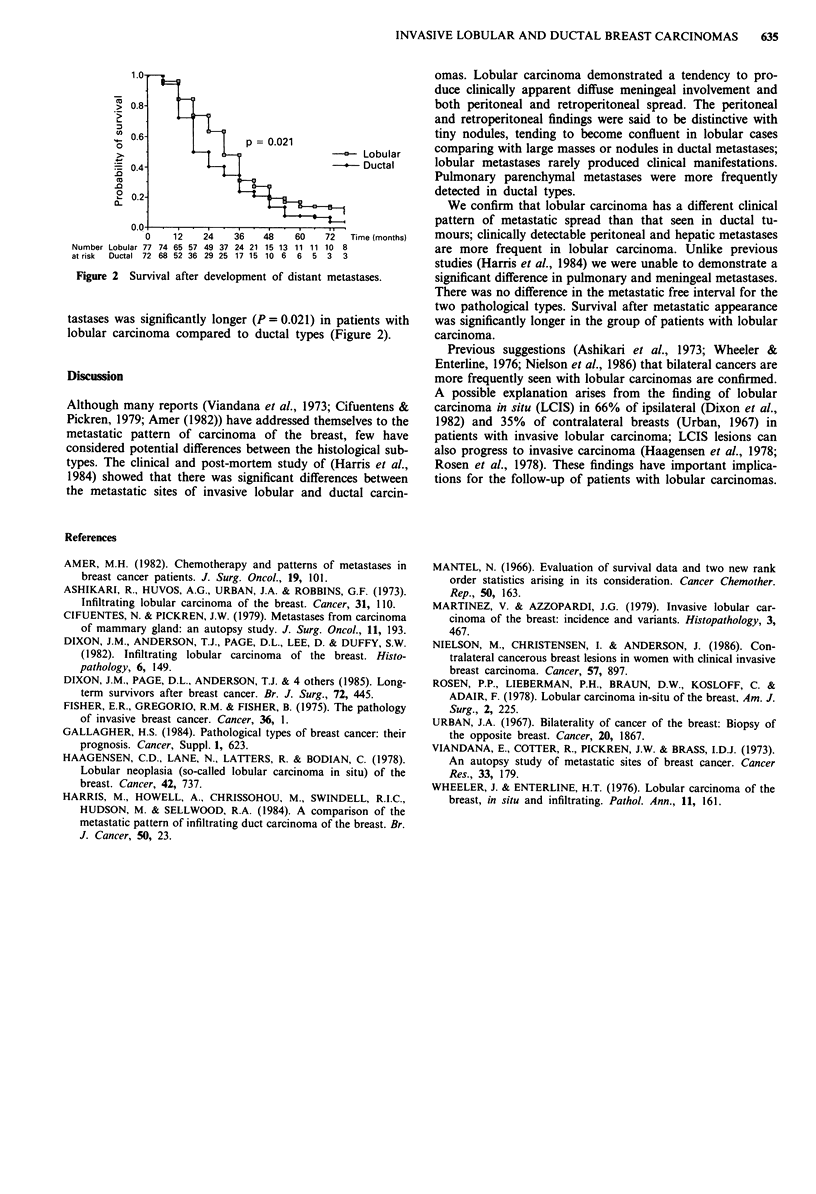# A comparison of the clinical metastatic patterns of invasive lobular and ductal carcinomas of the breast.

**DOI:** 10.1038/bjc.1991.145

**Published:** 1991-04

**Authors:** A. R. Dixon, I. O. Ellis, C. W. Elston, R. W. Blamey

**Affiliations:** City Hospital, Nottingham, UK.

## Abstract

Seventy seven patients with metastases from an invasive lobular carcinoma of the breast have been compared with 72 consecutive metastatic ductal carcinomas. There was no difference in the metastatic free interval between the two groups. A distinct pattern of clinical presentation of metastases was seen; hepatic (P = 0.01) and peritoneal metastases (P = 0.0003) occurred more commonly in lobular tumours. Bilateral cancers were more common in the lobular group (P = 0.01). No difference was seen in terms of meningeal and pulmonary metastases. Survival after metastases was significantly longer in patients with metastatic lobular carcinoma (P = 0.02).


Br. J. Cancer (1991), 63, 634–635
© Macmillan Press Ltd., 1991

### A comparison of the clinical metastatic patterns of invasive lobular and ductal carcinomas of the breast

A.R. Dixon, I.O. Ellis, C.W. Elston & R.W. Blamey

*City Hospital, Hucknall Road, Nottingham, NG5 1PB, UK.*

**Summary** Seventy seven patients with metastases from an invasive lobular carcinoma of the breast have been compared with 72 consecutive metastatic ductal carcinomas. There was no difference in the metastatic free interval between the two groups. A distinct pattern of clinical presentation of metastases was seen; hepatic ($P = 0.01$) and peritoneal metastases ($P = 0.0003$) occurred more commonly in lobular tumours. Bilateral cancers were more common in the lobular group ($P = 0.01$). No difference was seen in terms of meningeal and pulmonary metastases. Survival after metastases was significantly longer in patients with metastatic lobular carcinoma ($P = 0.02$).

Differences in cell morphology, growth patterns and tissue response allow for the division of invasive adenocarcinomas of the breast into specific types (Gallagher, 1984); infiltrating ductal (80%) and lobular (8–14.7%) carcinomas are the commonest (Martinez & Azzopardi, 1979; Dixon *et al.*, 1982). The largest sub-group (65–68%) show no specific characteristics and have been termed carcinoma NOS — not otherwise specified (Fisher *et al.*, 1975) or ductal NOS (Dixon *et al.*, 1985). These histological differences have been shown to have important influences upon prognosis (Gallagher, 1984; Dixon *et al.*, 1985). A study based on clinical and post-mortem material (Harris *et al.*, 1984) showed that lobular carcinoma of the breast had a distinctly different metastatic pattern to that seen in ductal carcinomas: lobular types were associated with carcinomatous meningitis and both peritoneal and retroperitoneal metastases of a diffuse micro-nodular fashion; lung parenchymal metastases were more common with ductal tumours. This series of lobular carcinomas has been studied in an attempt to verify this observation.

### Patients and methods

One thousand patients with primary operable breast cancer were treated in the Breast Unit at the City Hospital, Nottingham, between October 1973 and March 1983. Treatment consisted of simple mastectomy, subcutaneous mastectomy or lumpectomy followed by irradiation; no patient received adjuvant systemic therapy. Using established criteria (Martinez & Azzopardi, 1979) two pathologists (I.O.E. and C.W.E.) classified 118 of these as invasive lobular carcinomas. These case notes were reviewed and the following data obtained: metastatic free interval, site of metastases and survival after development of metastases. Distant metastatic involvement was recorded as that which was determined clinically during follow-up and verified by the relevant investigation. Port-mortem studies were not carried out. Seventy seven patients (65%) with lobular tumours developed distant metastases. These patients were compared with 72 consecutive metastatic ductal carcinomas that developed in the first 134 patients of the Nottingham series.

Life table analysis was used to compare survival between the two groups after the development of metastases. The $\chi^2$ method was applied to determine statistical differences between two curves (Mantel, 1966). Fisher's exact test was used to determine statistical differences between sites of metastases.

Correspondence: A.R. Dixon, Professorial Department of Surgery, City Hospital, Nottingham, NG5 1PB, UK.
Received and accepted 19 June 1990.

### Results

The median age at development of metastases was 56 years (28–72 years) for invasive lobular tumours and 54 years (37–70 years) for ductal carcinomas (not significant); medial follow-up time for both groups was 72 months. The sites of distant metastases as diagnosed clinically are shown in Table I. Hepatic ($P = 0.01$) and peritoneal (0.0003) metastases were detected significantly more often in lobular carcinomas. The clinical presentation of the 14 lobular peritoneal metastases included: retroperitoneal ureteric obstruction (3), large bowel obstruction and perforation (1), 'linitis plastica' of the stomach (2), small bowel obstruction (3), omental masses (5). The six patients with obstructive/dysphagic symptoms underwent laparotomy, the operative findings in each case being tiny serosal nodules, widespread throughout the bowel. These nodules were confluent at the site of obstruction.

No significant difference existed between the two pathological types in terms of skeletal, pulmonary, pleural and cerebral metastases. Eighteen of the 149 patients with metastases developed metachronous contralateral cancers (12%), 14 of these occurred in the lobular group ($P = 0.01$).

Although there was no difference in the metastatic free interval (Figure 1), survival from diagnosis of systemic me-

**Table I** Clinical metastatic pattern of 77 invasive lobular carcinomas compared with 72 invasive ductal carcinomas

| | *Lobular (%)* | *Ductal (%)* | *P* |
|---|---|---|---|
| Lung | 18 (24) | 16 (22) | NS |
| Pleural | 48 (63) | 56 (78) | NS |
| Bone | 46 (60) | 52 (72) | NS |
| Liver | 27 (35) | 13 (18) | 0.01 |
| Peritoneum | 14 (18) | 1 ( 1) | 0.0003 |
| Salivary gland | 2 ( 3) | 0 ( 0) | NS |
| Brain parenchyma | 1 ( 1) | 3 ( 4) | NS |
| Leptomeninges | 1 ( 1) | 2 ( 3) | NS |
| Opposite breast | 14 (18) | 4 ( 6) | 0.01 |

**Figure 1** Distant metastatic free interval.

**Figure 2** Survival after development of distant metastases.

tastases was significantly longer ($P = 0.021$) in patients with lobular carcinoma compared to ductal types (Figure 2).

### Discussion

Although many reports (Viandana *et al.*, 1973; Cifuentens & Pickren, 1979; Amer (1982)) have addressed themselves to the metastatic pattern of carcinoma of the breast, few have considered potential differences between the histological subtypes. The clinical and post-mortem study of (Harris *et al.*, 1984) showed that there was significant differences between the metastatic sites of invasive lobular and ductal carcinomas. Lobular carcinoma demonstrated a tendency to produce clinically apparent diffuse meningeal involvement and both peritoneal and retroperitoneal spread. The peritoneal and retroperitoneal findings were said to be distinctive with tiny nodules, tending to become confluent in lobular cases comparing with large masses or nodules in ductal metastases; lobular metastases rarely produced clinical manifestations. Pulmonary parenchymal metastases were more frequently detected in ductal types.

We confirm that lobular carcinoma has a different clinical pattern of metastatic spread than that seen in ductal tumours; clinically detectable peritoneal and hepatic metastases are more frequent in lobular carcinoma. Unlike previous studies (Harris *et al.*, 1984) we were unable to demonstrate a significant difference in pulmonary and meningeal metastases. There was no difference in the metastatic free interval for the two pathological types. Survival after metastatic appearance was significantly longer in the group of patients with lobular carcinoma.

Previous suggestions (Ashikari *et al.*, 1973; Wheeler & Enterline, 1976; Nielson *et al.*, 1986) that bilateral cancers are more frequently seen with lobular carcinomas are confirmed. A possible explanation arises from the finding of lobular carcinoma *in situ* (LCIS) in 66% of ipsilateral (Dixon *et al.*, 1982) and 35% of contralateral breasts (Urban, 1967) in patients with invasive lobular carcinoma; LCIS lesions can also progress to invasive carcinoma (Haagensen *et al.*, 1978; Rosen *et al.*, 1978). These findings have important implications for the follow-up of patients with lobular carcinomas.

### References

AMER, M.H. (1982). Chemotherapy and patterns of metastases in breast cancer patients. *J. Surg. Oncol.*, **19**, 101.

ASHIKARI, R., HUVOS, A.G., URBAN, J.A. & ROBBINS, G.F. (1973). Infiltrating lobular carcinoma of the breast. *Cancer*, **31**, 110.

CIFUENTES, N. & PICKREN, J.W. (1979). Metastases from carcinoma of mammary gland: an autopsy study. *J. Surg. Oncol.*, **11**, 193.

DIXON, J.M., ANDERSON, T.J., PAGE, D.L., LEE, D. & DUFFY, S.W. (1982). Infiltrating lobular carcinoma of the breast. *Histopathology*, **6**, 149.

DIXON, J.M., PAGE, D.L., ANDERSON, T.J. & 4 others (1985). Long-term survivors after breast cancer. *Br. J. Surg.*, **72**, 445.

FISHER, E.R., GREGORIO, R.M. & FISHER, B. (1975). The pathology of invasive breast cancer. *Cancer*, **36**, 1.

GALLAGHER, H.S. (1984). Pathological types of breast cancer: their prognosis. *Cancer*, Suppl. **1**, 623.

HAAGENSEN, C.D., LANE, N., LATTERS, R. & BODIAN, C. (1978). Lobular neoplasia (so-called lobular carcinoma in situ) of the breast. *Cancer*, **42**, 737.

HARRIS, M., HOWELL, A., CHRISSOHOU, M., SWINDELL, R.I.C., HUDSON, M. & SELLWOOD, R.A. (1984). A comparison of the metastatic pattern of infiltrating duct carcinoma of the breast. *Br. J. Cancer*, **50**, 23.

MANTEL, N. (1966). Evaluation of survival data and two new rank order statistics arising in its consideration. *Cancer Chemother. Rep.*, **50**, 163.

MARTINEZ, V. & AZZOPARDI, J.G. (1979). Invasive lobular carcinoma of the breast: incidence and variants. *Histopathology*, **3**, 467.

NIELSON, M., CHRISTENSEN, I. & ANDERSON, J. (1986). Contralateral cancerous breast lesions in women with clinical invasive breast carcinoma. *Cancer*, **57**, 897.

ROSEN, P.P., LIEBERMAN, P.H., BRAUN, D.W., KOSLOFF, C. & ADAIR, F. (1978). Lobular carcinoma in-situ of the breast. *Am. J. Surg.*, **2**, 225.

URBAN, J.A. (1967). Bilaterality of cancer of the breast: Biopsy of the opposite breast. *Cancer*, **20**, 1867.

VIANDANA, E., COTTER, R., PICKREN, J.W. & BRASS, I.D.J. (1973). An autopsy study of metastatic sites of breast cancer. *Cancer Res.*, **33**, 179.

WHEELER, J. & ENTERLINE, H.T. (1976). Lobular carcinoma of the breast, *in situ* and infiltrating. *Pathol. Ann.*, **11**, 161.